# Uncommon structural and bonding properties in Ag_16_B_4_O_10_†

**DOI:** 10.1039/c9sc05185f

**Published:** 2019-12-09

**Authors:** Anton Kovalevskiy, Congling Yin, Jürgen Nuss, Ulrich Wedig, Martin Jansen

**Affiliations:** Max-Planck-Institut für Festkörperforschung Heisenbergstr. 1 70569 Stuttgart Germany m.jansen@fkf.mpg.de; MOE Key Laboratory of New Processing Technology for Nonferrous Metal and Materials, Guangxi Key Laboratory of Optical and Electronic Materials and Devices, College of Materials Science and Engineering, Guilin University of Technology Guilin 541004 P. R. China congling.yin@glut.edu.cn

## Abstract

Ag_16_B_4_O_10_ has been obtained as a coarse crystalline material *via* hydrothermal synthesis, and was characterized by X-ray single crystal and powder diffraction, conductivity and magnetic susceptibility measurements, as well as by DFT based theoretical analyses. Neither composition nor crystal structure nor valence electron counts can be fully rationalized by applying known bonding schemes. While the rare cage anion (B_4_O_10_)^8−^ is electron precise, and reflects standard bonding properties, the silver ion substructure necessarily has to accommodate eight excess electrons per formula unit, (Ag^+^)_16_(B^3+^)_4_(O^2−^)_10_ × 8e^−^, rendering the compound sub-valent with respect to silver. However, the phenomena commonly associated with sub-valence metal (partial) structures are not perceptible in this case. Experimentally, the compound has been found to be semiconducting and diamagnetic, ruling out the presence of itinerant electrons; hence the excess electrons have to localize pairwise. However, no pairwise contractions of silver atoms are realized in the structure, thus excluding formation of 2e–2c bonds. Rather, cluster-like aggregates of an approximately tetrahedral shape exist where the Ag–Ag separations are significantly smaller than in elemental silver. The number of these subunits per formula is four, thus matching the required number of sites for pairwise nesting of eight excess electrons. This scenario has been corroborated by computational analyses of the densities of states and electron localization function (ELF), which clearly indicate the presence of an attractor within the shrunken tetrahedral voids in the silver substructure. However, one bonding electron pair of s and p type skeleton electrons per cluster unit is extremely low, and the significant propensity to form and the thermal stability of the title compound suggest d^10^–d^10^ bonding interactions to strengthen the inter-cluster bonding in a synergistic fashion. With the present state of knowledge, such a particular bonding pattern appears to be a singular feature of the oxide chemistry of silver; however, as indicated by analogous findings in related silver oxides, it is evolving as a general one.

## Abstract 2

Ag_16_B_4_O_10_, obtained *via* hydrothermal synthesis, displays an unprecedented bonding scheme, hosting excess electrons localized pairwise in cluster-like silver subunits.

## Graphical abstract

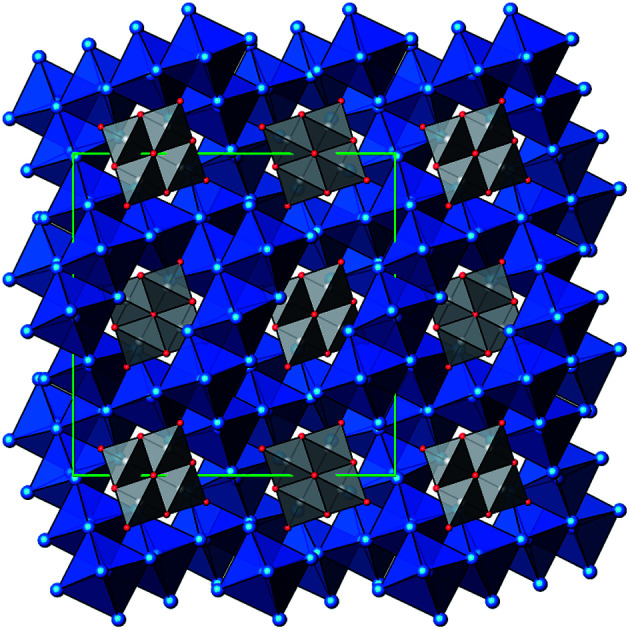

## Introduction

Employing advanced techniques in chemical synthesis constitutes an effective approach to realizing unconventional compounds, sometimes opening access even to new classes of materials featuring, *e.g.*, novel bonding principles. One such example concerns multinary silver oxides, which are thermally notoriously labile. Using specially designed Bridgeman-type autoclaves made of distinctly scaling-resistant steel,^1^ enduring conditions applied of up to 7 × 10^8^ Pa pressure of oxygen and up to 973 K temperature enables suppression of the thermal degradation of Ag_2_O and thus reaction of this oxide in all-solid state reactions with any other binary oxide in the periodic table. In a preconceived view, one would expect the oxides attainable this way, Ag_*x*_M_*y*_O_*z*_ (M = nonmetal or metal), to represent analogues of respective alkali metal oxides. However, systematic exploration of such systems has revealed that singular early observations of conspicuously short Ag^+^–Ag^+^ separations in oxides are not strange exceptions, but manifestations of a general feature of the chemistry of silver.^2–4^ Primarily in oxides with high silver contents, silver(i) ions tend to aggregate forming partial structures that are topologically reminiscent of elemental silver. Furthermore, such structural motifs are associated with specific physical properties.^5–7^ From these findings we concluded thus: “The substructures thereby formed have empty s and p conduction bands, which can easily accommodate further electrons on reduction”.^3^ As a consequence, one would expect oxides to exist that contain silver in oxidation states between 0 and +1, *i.e.* sub-valent silver. Indeed, a few candidates fulfilling such an expectation were communicated, *e.g.* Ag_3_O^8^ and Ag_5_GeO_4_.^9–11^ Here we report on a new compound, Ag_16_B_4_O_10_, a rather exotic oxide containing nominally Ag^0.5+^, or 8 excess electrons when assigning standard oxidation states according to (Ag^+^)_16_(B^3+^)_4_(O^2−^)_10_ × 8e^−^. Interestingly, the cation substructure Ag_16_B_4_ corresponds to the *ccp* arrangement of metallic silver, where out of every 20 silver atoms four adjacent ones, forming a tetrahedron, are replaced by boron atoms. In turn, the latter are coordinated by four oxygen atoms each, resulting in the rare adamantane related cage anion B_4_O_10_^8−^,^12^ for which precise dimensions have been determined here for the first time, unaffected by disorder^13^ or under-occupation of atomic positions.^14^ The findings shed light on the common effects of covalent bond length contractions caused by multiple bonding and/or superimposed polar contributions. Comparison with isosteric P_4_O_10_ reveals that the bond distances to the terminal oxygen atoms are significantly less contracted in the borate, which does not feature any low lying orbitals that might mediate multiple bonding, while in accordance with the small difference in the electronegativities^15^ of boron (2.051) and phosphorus (2.253), possible contractions due to polarities of the B–O and P–O terminal bonds are of comparable magnitudes. The strange overall composition encountered is even more puzzling since such a kind of ternary oxide, a silver borate, is expected to follow heuristic rules of stoichiometry. In the light of the electron counts given, which suggest the presence of itinerant excess electrons, it comes as a surprise that according to resistivity measurements the title compound is a small band gap semiconductor. However, analyses of electron localizations using DFT based calculations comply with the experimental results. Apparently, a new generalizable facet of the chemistry of silver becomes recognizable, which will complement text book knowledge of this element and bears potential for realizing interesting material properties due to the subtleties of electron localizations found.

## Experimental

### Synthetic procedures

Polycrystalline samples of Ag_16_B_4_O_10_ were prepared by the solid state reaction of elemental Ag and H_3_BO_3_, or B_2_O_3_, in stainless-steel autoclaves at elevated oxygen pressures of 2 × 10^7^ to 5 × 10^7^ Pa and temperatures of 563–623 K. The starting materials were intimately mixed, pressed to pellets and placed in gold tubes, which were sealed at one end and crimped at the other. Small amounts of H_2_O were added into the gold tubes to promote crystallization. In a typical synthesis batch 0.03 mol of Ag and 0.01 mol of H_3_BO_3_ were used, with the addition of 0.5 mL H_2_O.

### X-ray powder diffraction

**Fig. 1:**
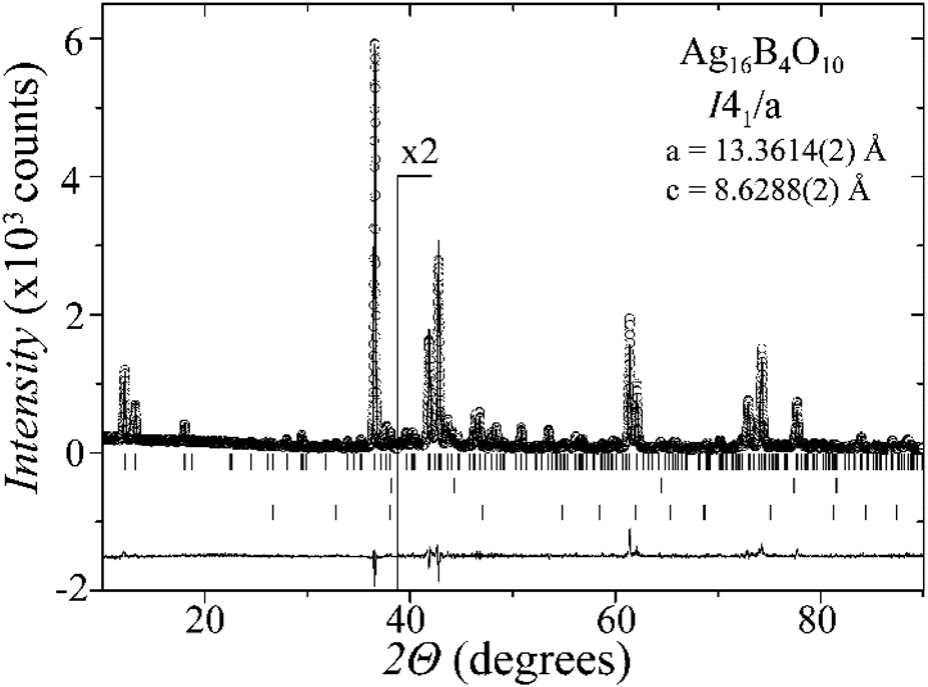
PXRD pattern of the Ag_16_B_4_O_10_ sample, showing the observed (circles), Rietveld fit (black line) and difference curve (gray line). The upper, middle and bottom bars mark the reflections for Ag_16_B_4_O_10_, Ag and Ag_2_O, respectively. The intensity is doubled in the inset for clarity.

Laboratory powder X-ray diffraction (PXRD) data (Fig. 1) were collected at room temperature on a Bruker D8 diffractometer with germanium monochromatized Cu-Kα1 radiation (*λ* = 1.5406 Å), in steps of 0.01° over a 2*θ* range of 10–90 degrees. A Rietveld profile fit was carried out with the TOPAS-4.2.0.2 (AXS) program.^16^ The refined parameters were scale factor, sample displacement (mm), background as a Chebyshev polynomial of 5th degree, 1/*x* function, crystallite size, micro-strain (Stephens broaden model^17^) and cell constants. In addition, temperature dependent PXRD data were collected in the temperature range from room temperature to 773 K, in intervals of 50–100 K, as shown in the ESI, Fig. S1.†

### X-ray single-crystal diffraction

A crystal suitable for single-crystal X-ray diffraction was selected in a drybox (M. Braun, Garching, Germany) under an argon atmosphere (<0.1 ppm O_2_, H_2_O) and mounted in a sealed glass capillary. Diffraction data were collected at room temperature (298 K) with a SMART APEX-I CCD X-ray diffractometer (Bruker AXS, Karlsruhe, Germany), using Mo-Kα radiation. The intensities of the Bragg reflections were integrated with the SAINT subprogram in the Bruker Suite software.^18^ A multi-scan absorption correction was applied using SADABS.^19^ The crystal structures were solved by direct methods and refined by full-matrix least-square fitting with the SHELXTL software package.^20,21^ Experimental details and crystallographic data are given in Tables S1–S3.†

### Resistivity and magnetic susceptibility measurement

Magnetic properties were studied using a Quantum Design MPMS SQUID Magnetometer. Zero-field-cooled (ZFC) and field-cooled (FC) magnetic susceptibility data were recorded in a 10 000 Oe field while warming the sample from 5 to 300 K. Resistivities of polycrystalline bars (approximate dimensions 3 × 3 × 10 mm^3^) of Ag_16_B_4_O_10_ were recorded using a standard four-probe dc technique on a Quantum Design physical property measurement system.

### Computational methods

Density functional (DFT) calculations, based on the experimental structure of Ag_16_B_4_O_10_, were performed using the CRYSTAL17 program package.^22^ The bands in the semi-core and valence space, considering 19 valence electrons of each silver atom, 6 for O and 3 for B, are expanded in terms of local Gaussian basis functions. The core electrons are represented by scalar relativistic pseudopotentials. Details on the exponents and contractions are given in the ESI.† The integration in reciprocal space was based on 242 *k*-points in the irreducible part of the Brillouin zone. The results presented here are obtained with the short-range-separated hybrid functional HSEsol^23^ for the exchange and correlation terms in the Kohn–Sham equations. Atomic charges were evaluated by analyzing the electron density topologically according to the QTAIM approach.^24^ A search of the atomic basins was performed with the critic2 (ref. 25 and 26) program on the basis of a 321 × 321 × 201 grid of data points of the valence electron density, augmented by core densities. Within the basins, the valence density was integrated in order to get the net charges. Data grids of the electron localization function^27,28^ (ELF) were computed with TOPOND,^29^ integrated in CRYSTAL17. Structural data and volumetric data were visualized with the VESTA code.^30^

## Results and discussion

A new compound, Ag_16_B_4_O_10_, has been obtained as a coarse crystalline material *via* solid state synthesis. The shiny black crystallites are insensitive to humid air and start decomposing thermally at ∼623 K with silver metal and amorphous B_2_O_3_ resulting in the final solid residues, see Fig. S1.† The title compound can be prepared from various starting materials in the required molar ratios, as there are boron(iii) oxide, boronic acid, silver oxide and finally elemental silver as an essential component, while adding varying amounts of water as a mineralizing agent. At first glance it appears unintuitive that for the synthesis of such a considerably reduced material applying moderately elevated oxygen pressures of 2 × 10^7^ to 5 × 10^7^ Pa is an indispensable requirement. However, running the experiments at ambient pressure results in the decomposition of silver oxide to the metal, while a too high oxygen pressure would end up in the formation of silver(i) borates. So, the synthesis of the title compound is a delicate balancing act, and even for the optimized synthesis conditions, given above in detail, one or the other synthesis run may fail in yielding single phase products. Fig. 1 displays a Rietveld profile fit of an X-ray powder diffractogram of a sample obtained using the optimized synthesis procedure; a three-phase refinement has revealed that only traces of Ag (2.1%) and Ag_2_O (2.3%) are present. The atomic parameters obtained are given in Table S4.†

**Fig. 2:**
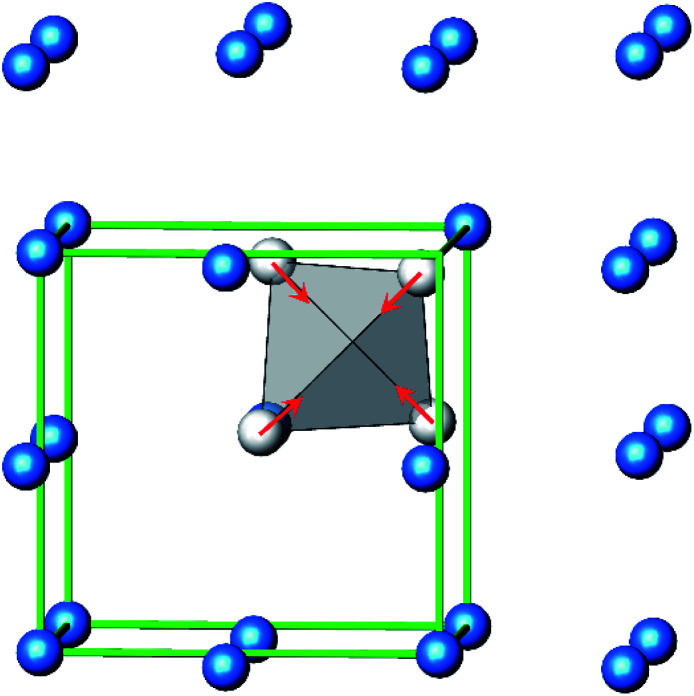
Schematic presentation of the cation substructure of Ag_16_B_4_O_10_, emphasizing its relationship to a *ccp* packing; Ag atoms (blue), partially replaced by B (grey).

**Fig. 3:**
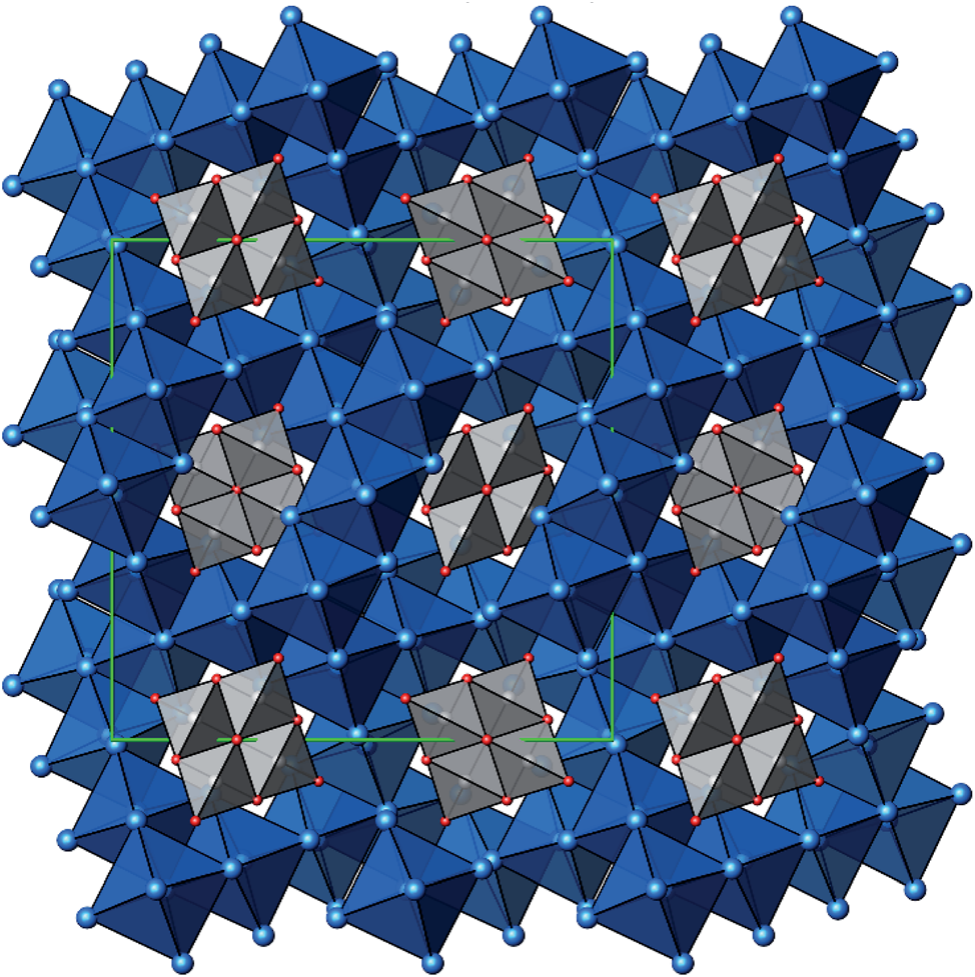
Projection of the crystal structure of Ag_16_B_4_O_10_, view along [001], with margins of the unit cell (green). Color code: Ag (blue spheres), B (grey spheres), O (red spheres), blue octahedra (Ag_6_), grey tetrahedra (BO_4_).

**Fig. 4:**
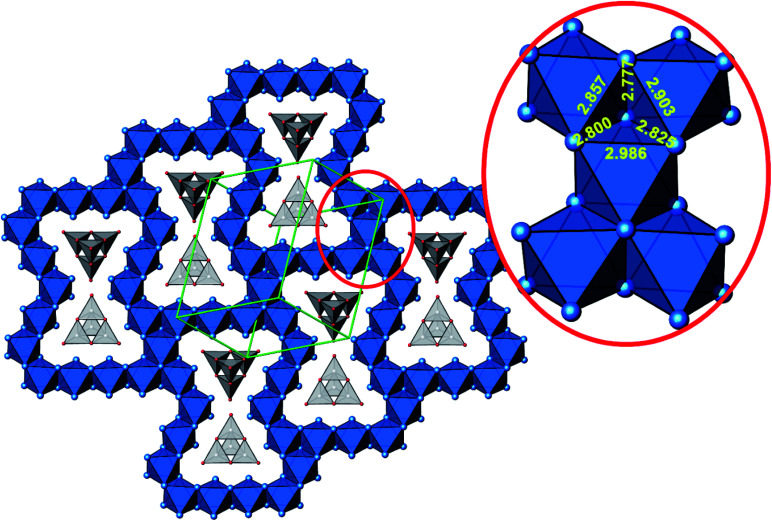
Cut-out of the crystal structure of Ag_16_B_4_O_10_, view along [121]. Magnification (red circle) highlights a block consisting of five edge sharing octahedra with Ag–Ag distances labeled. Same color code as in Fig. 3.

The constitution has been confirmed unambiguously by single crystal X-ray structure determination. As illustrated in Fig. 2, the general structural organization of Ag_16_B_4_O_10_ derives straight forwardly from a *ccp* pattern of elemental silver: out of every twenty silver atoms four adjacent ones are replaced by boron. Since boron atoms are of considerably smaller size compared to silver, the substitution generates some empty space tolerating the insertion of oxygen atoms to form the polyoxoanion B_4_O_10_^8−^. From this picture it is immediately obvious that the title compound ideally matches the notion of classifying crystal structures of extended inorganic solids rather based on the packing of the cations^31^ than on that of anions. Even beyond, it lends strong support to the further reaching concept according to which certain oxides may be regarded as alloys being stuffed with oxygen.^32,33^ This particular structural interrelation is underpinned by Fig. 3 and 4, showing the crystal structure of the title compound along the view directions [001] and [121], respectively.

In more detail, all silver atoms are engaged in octahedral homoatomic building units, which are linked by sharing edges and vertices to form a 3D framework. Every silver atom is coordinated by either one or two oxygen atoms at a distance typical of this pair of atoms *d*(Ag–O) = 2.20–2.35 Å (see Table S3†).

The rare complex anion B_4_O_10_^8−^ has been observed here as an “isolated”, *i.e.* unbridged, entity for the first time.^12^ Two previous reports present this building block as part of an extended framework. Moreover, in both cases the structural analyses performed suffer from disorder, where occupation factors of 1/2 for the intra-cage bridging oxygen atoms even question the connectivity, *i.e.* the presence of such an integral anion, at all,^13^ or from refining split atom positions for the anion in two orientations, impairing the accuracy of the data obtained.^14^

**Fig. 5:**
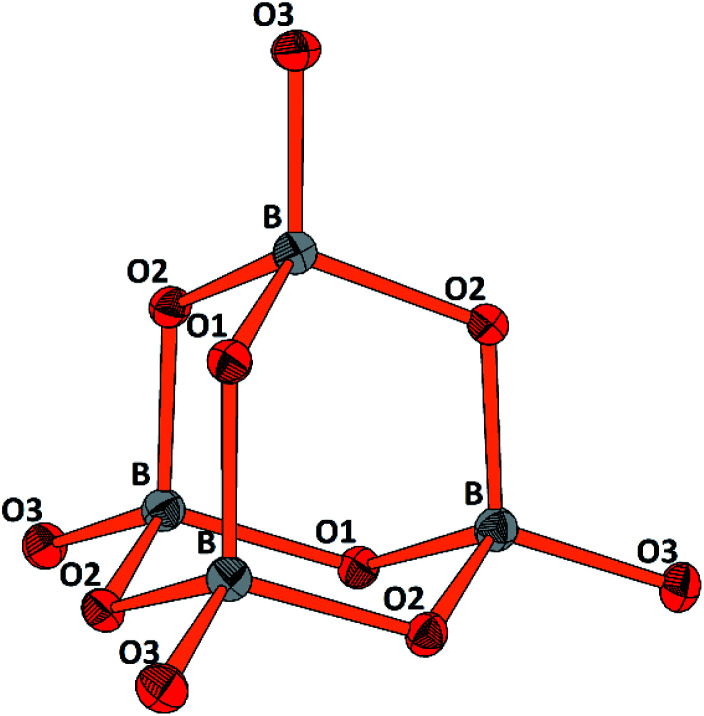
Perspective representation of the B_4_O_10_^8−^ anion. Color code: B (grey), O (red), displacement ellipsoids drawn at the 50% probability level. For the labeling scheme *c.f.* Tables S2 and S3.†

The molecular anion, see Fig. 5, is isostructural and (valence) isoelectronic to P_4_O_10_. The bond lengths are in the expected range, see Tables 1 and S3,† and the variations reflect the position of the oxygen atoms, terminal or bridging within the adamantane type of cage. Drawing the chemical bonds of the cage anion in terms of the Lewis concept requires placing a formal negative charge on both, the boron and the terminal oxygen atoms. Although a formalism, this tells that the bonding situation is special and suggests looking for the respective structural distinguishing features by comparing with the isosteric cage molecule P_4_O_10_. Such an approach would imply comparison of the bonding of elements from the second and third row of the periodic table. For the latter, contributions of d_π_–p_π_ multiple bonding and superimposed polar interactions are factors of influence,^34^ while for the former such d_π_–p_π_, or p_π_–p_π_ interactions are not relevant; consequently, the bonding within the borate anion, where all boron atoms are in tetrahedral coordination, has to be analyzed exclusively in terms of σ bonding schemes. Indeed, comparing the bond lengths in B_4_O_10_^8−^ and P_4_O_10_ (ref. 35) reveals significant differences. In the borate the terminal B–O bonds are only 4.4% shorter than the bridging ones, whereas the respective shrinkage amounts to 10.1% for the phosphorous oxide, see Table 1. Obviously, for the P–O_terminal_ bonds multiple bonding and polarity act in a synergistic fashion, while in the borate for the shrinkage of the B–O_terminal_ bonds only polar inter-actions superimposing the σ bond are relevant, resulting in a smaller net effect.

**Table 1:** Comparison of averaged bridging (br) and terminal (tr) B–O, P–O bonds/Å and O–B–O, O–P–O angles/° in [B_4_O_10_]^8−^ and P_4_O_10_

[B_4_O_10_]^8−^		P_4_O_10_	
B–O(tr)	1.431, *Δ* = −4.4%	P–O(tr)	1.432, *Δ* = −10.1%
B–O(br)	1.497	P–O(br)	1.591
O(tr)–B–O(br)	111.72	O(tr)–P–O (br)	116.06
O(br)–B–O(br)	107.13	O(br)–P–O (br)	102.06

**Fig. 6:**
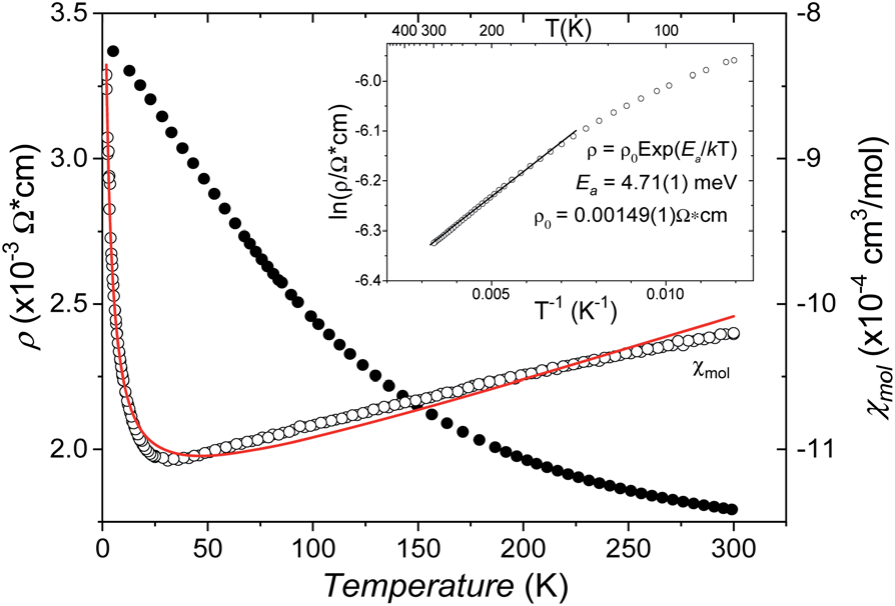
The temperature dependent resistivity (filled circles) and mole susceptibility (open circles). The red line shows the three-term fit (see the text) of susceptibility. The inset shows the Arrhenius plot of resistivity.

The B_4_O_10_^8−^ cage like polyoxoanion is electron precise, and thus the excess electrons according to (Ag^+^)_16_(B^3+^)_4_(O^2−^)_10_ × 8e^−^ have to be accommodated by the silver substructure. As a consequence, one would expect itinerant electrons being present, giving rise to metallic conductivity. Surprisingly, the compound is a small band gap semiconductor, and diamagnetic, as shown in Fig. 6. By fitting the resistivities in the high temperature range above 150 K to the Arrhenius equation (Fig. 6 inset), an experimental band gap of 4.71(1) meV was obtained. This value is essentially identical to the calculated one, see below. The diamagnetic susceptibilities are estimated to be −1147 × 10^−6^ cm^3^ mol^−1^, based on an analysis of the magnetic susceptibilities applying a Curie–Weiss, Pauli and diamagnetic term. The upturn tail below 30 K is dominated by an unknown paramagnetic impurity (about 0.2% with *S* = 1/2). These results indicate the presence of localized and paired excess electrons. However, no particularly shortened individual Ag–Ag bond-length can be identified that would provide evidence for normal 2e–2c bonds, although the interatomic silver separations are on average smaller than those found for elemental silver. As can be seen from Fig. 4 (red circle) the ‘empty’ octahedral silver units appear to cluster, forming blocks consisting of five edge sharing octahedra. Each such cluster entity features two tetrahedral voids, where three out of six edges formed by silver atoms are substantially shorter (2.80 Å on average) than the separation in elemental silver (2.89 Å). This gives a first qualitative hint to the fact that these are the regions in real space where the excess electrons might be accommodated. Since these tetrahedral sites add up to four per formula unit, which in total may host four pairs of excess electrons, *i.e.* all of them, this view would comply with the experimentally found magnetic and transport properties. We exclude the possible presence of hydride anions, filling the contracted voids, for chemical reasons. Along any synthesis protocol yielding the title compound protons are essential constituents, and, moreover, applying an elevated oxygen pressure is an indispensable requirement. The pronouncedly basic and strongly reducing hydride ion cannot exist under such conditions.

**Fig. 7:**
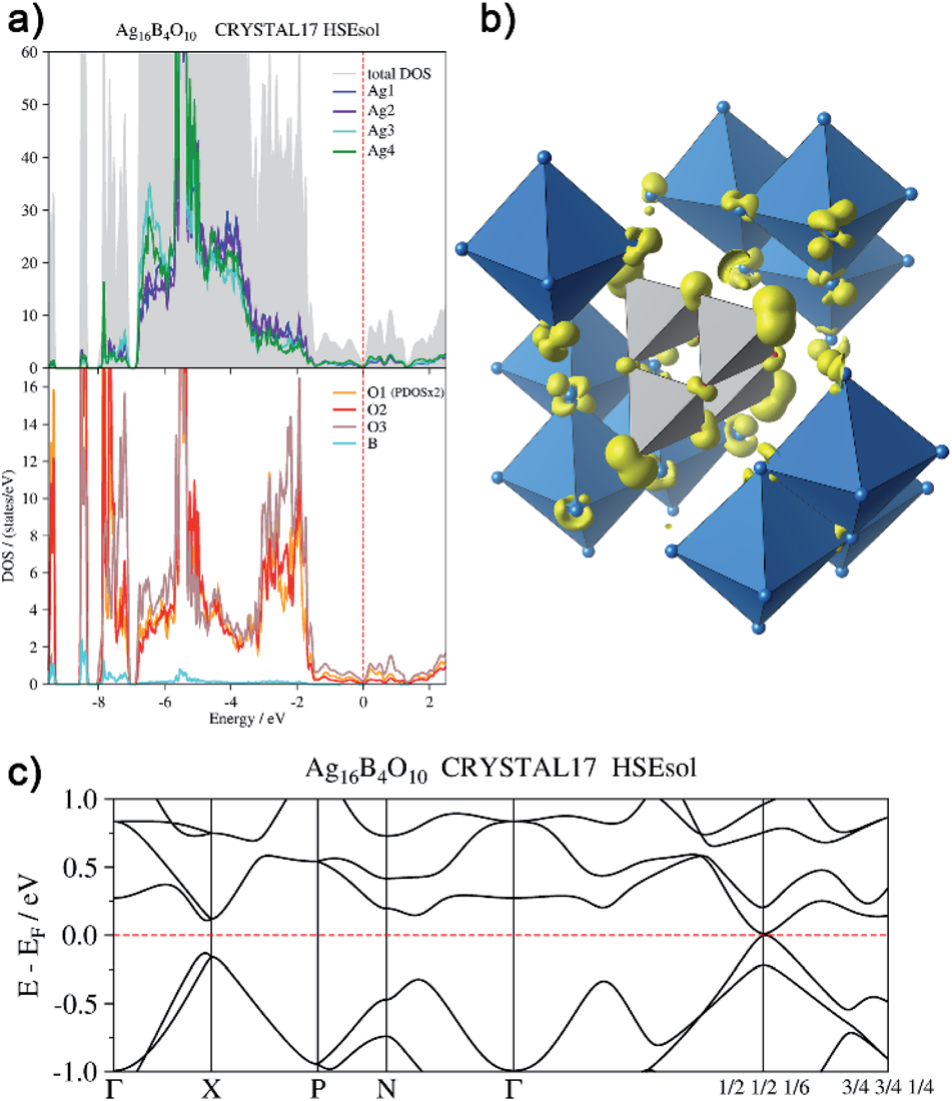
(a) Total and atom-projected density of states, (b) isosurface (0.04 e^−^ Å^−3^) of the electron density generated by the disperse bands below the Fermi level (*E*_F_ −1.44 eV to *E*_F_), (c) bands at *E*_F_.

In order to back this explanation quantitatively, we performed a DFT based computational analysis on the density of states (DOS), electron densities and localizations. DOS as well as the projections onto the atoms (PDOS) is shown in Fig. 7a. Over the whole energy range, which is shifted relative to the Fermi level (*E*_F_ = 0 eV), oxygen basis functions contribute to the DOS. Below −7 eV, the silver PDOS is low. Bands are flat and can be associated with the B–O bonds in the B_4_O_10_^8−^ unit. The low contribution of boron to these bands points to the ionic character of the B–O bonds. The DOS between −7 eV and −3.2 eV is dominated by the 4d states of the silver atoms, however, it shows two peculiarities. At lower energies within this range, Ag3 and Ag4 show a significantly higher PDOS than Ag1 and Ag2, due to additional 5s-contributions. At −5.5 eV, the DOS shows a maximum. Here further flat bands attributed to B–O bonds can be assigned within the manifold of silver d-states, in this case, however, with a higher Ag 4d ratio. In the range between −3.2 eV and −1.4 eV the PDOS of silver and oxygen atoms contribute at nearly equal weight to the total DOS. These bands are related to the interaction between the oxygen lone pairs and the silver 4d-shells.

From −1.44 eV up to the Fermi level bands with higher dispersion are located. The respective atomic contributions can be visualized by drawing isosurfaces of the electron density computed from these bands (Fig. 7b). These bands are delocalized over the whole crystal. Note, for clarity reasons, only a cut-out of the isosurface is shown in Fig. 7b. The bands are filled and the system is not metallic. There is a distinct band gap in most parts of the Brillouin zone. Only at *k* = (1/2 1/2 1/6) it becomes as tiny as 15 meV (Fig. 7c). The highest valence band and the lowest conduction band exhibit a parabolic dispersion. Significant effects of spin–orbit coupling are not expected, as the Ag 4d-shell is practically filled and the 5d-orbitals are only affected indirectly. Moreover, the projected densities of states show the same character in both bands. The band gap at *k* = (1/2 1/2 1/6) can be seen only in calculations with hybrid functionals. When using gradient corrected (GGA) functionals without fractional Hartree–Fock exchange the band gap is underestimated and vanishes, although still a minimum of the DOS can be seen at *E*_F_.

The projections of the DOS shown in Fig. 7a are based on a partitioning with respect to atomic basis functions. In the linear expansion of the bands, a certain basis function may contribute to a state which is attributed to a neighboring atom, and an interpretation in terms of chemical bonding may not be unique. Additional methods in position space give complementary information. The partitioning of space resulting from a topological analysis of the total electron density^24^ gives atomic volumes and net charges, which are quite robust with respect to the choice of the basis set. The values computed for Ag_16_B_4_O_10_ are summarized in Table 2. The volumes and charges at the various atomic positions are clearly related to the connectivity. Ag3 and Ag4, which have a lower positive charge, have only one short distance <2.4 Å to the neighboring oxygen, whereas Ag1 and Ag2 with a higher positive charge have two of them. Likewise, the negative charge of the oxygen atoms varies, depending on whether they occupy a bridging or terminal position in the B_4_O_10_^8−^ unit. The high positive charge of the boron atom points to a substantially ionic character of the B–O bond.

**Table 2:** Atomic volumes and net charges obtained by a topological analysis of the total electron density^24^ (QTAIM)

	Volume/Å^3^	Net charge	Connectivity
Ag1	16.639	+0.354	Two nearest oxygen neighbors
Ag2	16.619	+0.344	
Ag3	17.151	+0.212	One nearest oxygen neighbor
Ag4	17.323	+0.236	
O1	10.217	−1.411	Bridging
O2	10.283	−1.438	
O3	11.363	−1.293	Terminal
B	1.533	+2.290	

**Fig. 8:**
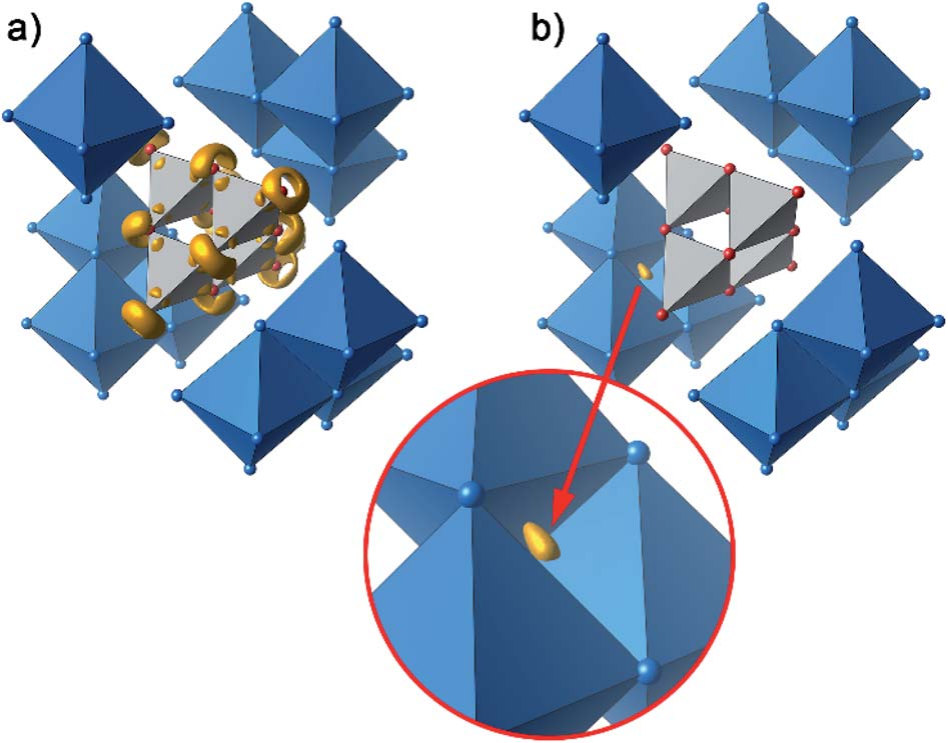
Selected domains of the electron localization function (ELF): (a) domains in the B_4_O_10_^8−^ anion with *η* = 0.84, (b) domain with *η* = 0.23 around the attractor in the tetrahedral voids.

Further information on the bonding characteristics in position space can be obtained by the inspection of the electron localization function^27,28^ (ELF), which can take values between 0 and 1. High ELF values *η* are related to atomic shells, covalent bonds and lone pairs. The ELF in Ag_16_B_4_O_10_ comprises three distinct features. At first, there are nearly spherical domains around each silver atom, representing the filled 4d^10^ shells. In Fig. 8, these domains are omitted for clarity reasons.

The domains associated with the B_4_O_10_^8−^ anion are represented in Fig. 8a showing the lone pairs of the oxygen ions and one attractor respectively along each B–O bond. The fact that the attractor is close to the oxygen atom is another hint to the polar character of this bond. A special feature of the ELF, an attractor at *η* = 0.23 appears in each tetrahedral void of the silver network. Fig. 8b shows the domain with *η* = 0.23 around one of them. *η* = 0.23 is a rather low value, though it represents a clear maximum in the ELF. Attractors with low ELF values may originate from the influence of close-by filled d-shells,^36^ nevertheless pointing to covalent interactions in the valence shell. Indeed, the face of the tetrahedron closest to the attractor contains two of the shortest Ag–Ag bonds (<2.80 Å) within the range from 2.78 Å to 3.23 Å of all Ag–Ag contacts (*cf.* metallic silver 2.89 Å).

Putting DOS, QTAIM and ELF information together, the bonding properties in Ag_16_B_4_O_10_ can be sketched as follows. The intra-molecular bonding in B_4_O_10_^8−^ is prevailingly polar in nature. The silver d-states span a wide energy range in the DOS pointing to significant d^10^–d^10^ interactions.^5–7^ Related to the sub-valence nature of the silver atoms, covalent interactions are apparent, especially in the tetrahedral voids of the silver clusters, where an ELF attractor is found. Thus, the silver network receives further stabilization beyond classical and d^10^–d^10^ bonding. Besides the obvious ionic interactions between the anionic unit and the silver framework, no indications for covalent contributions have been identified in the ELF. We understand the large overlap of the projected DOS of silver and oxygen to reflect dispersion interaction between the silver d^10^-shells and the oxygen lone pairs, both being highly polarizable, leading to a further stabilization of the compound.

## Conclusions

The new compound Ag_16_B_4_O_10_ displays singular compositional, structural, physical and electronic attributes. The structure can be described as a *ccp* arrangement of silver atoms, where four neighboring sites are substituted by boron. The packing of cations is stuffed with oxygen adjacent to the boron atoms, thus forming the rare [B_4_O_10_]^8−^ cage anion. Its geometry has been determined for the first time with high accuracy, not impaired by crystallographic bias as in two earlier reports.^13,14^ This has allowed us to assess the mechanisms (polarity of the bond *vs.* contributions of multiple bonding) behind shortening of the terminal bonds in comparison with isostructural and iso(valence)electronic species like P_4_O_10_. Since the polarities of the B–O and P–O bonds are comparable in magnitude, the significantly more pronounced shortening of the P–O bond indicates substantial O→P back donation *via* p_π_–d_π_ interactions, which are not possible in the borate. The borate cage anion is electron precise, thus the electron count based on conventional oxidation states, (Ag^+^)_16_(B^3+^)_4_(O^2−^)_10_ × 8e^−^, reveals an appreciable number of excess electrons to be accommodated in the silver sub-structure. Surprisingly, these excess electrons do not give rise to metallic conductivity, instead, small band gap semiconducting transport behavior was observed experimentally. Since moreover a diamagnetic response was recorded, the additional electrons have to be localized pairwise. Indeed, in the silver substructure, four significantly contracted tetrahedral voids per formula unit have been identified, which are suited to exactly accommodate the four pairs of excess electrons. DFT based theoretical analyses confirm opening of a small band gap and a significant ELF contour in the center of the contracted silver tetrahedra. In summary, our findings reported here are suited to consolidate earlier sparse reports on subvalent ternary silver oxides featuring similar structural and physical properties,^9–11^ characterized by compositions violating conventional rules of chemical valence with excess electrons localized in 2e-multicenter bonds in the silver substructure. The findings appear to reflect a singularity, so far only associated with silver, where in accordance with an early supposition^3^ d^10^–d^10^ bonding interactions provide extended Ag^+^ partial structures in position space involving low lying empty s and p states in reciprocal space. By deliberately accommodating excess electrons in those local s and p states, low electron count silver clusters are formed, stabilized by synergistic interactions among the filled 4d^10^-shells and local 2e-multicenter bonding of 5s and 5p skeleton electrons.

## Conflicts of interest

There are no conflicts to declare.

## Supplementary Material

SC-011-C9SC05185F-s001
