# Supplementary material for: Uncommon structural and bonding properties in Ag16B4O10
Source: Chem Sci. 2019 Dec 9;11(4):962–9. doi: 10.1039/c9sc05185f (PMC8145714; doi:10.1039/c9sc05185f)
Supplement: SC-011-C9SC05185F-s001 [file SC-011-C9SC05185F-s001.pdf]

Electronic Supplementary Material (ESI) for Chemical Science.
This journal is © The Royal Society of Chemistry 2019

# Supporting Information for

# Uncommon structural and bonding properties in $Ag_{16}B_4O_{10}$

Anton Kovalevskiy, [a] CongLingYin, [a,b,*] Jürgen Nuss, [a] Ulrich Wedig, [a] and Martin Jansen [a,*]

[a]Max-Planck-Institut für Festkörperforschung, Heisenbergstr. 1, 70569 Stuttgart, Germany

[b] MOE Key Laboratory of New Processing Technology for Nonferrous Metal and Materials, Guangxi Key Laboratory of Optical and Electronic Materials and Devices, College of Materials Science and Engineering, Guilin University of Technology, Guilin 541004 P. R. China

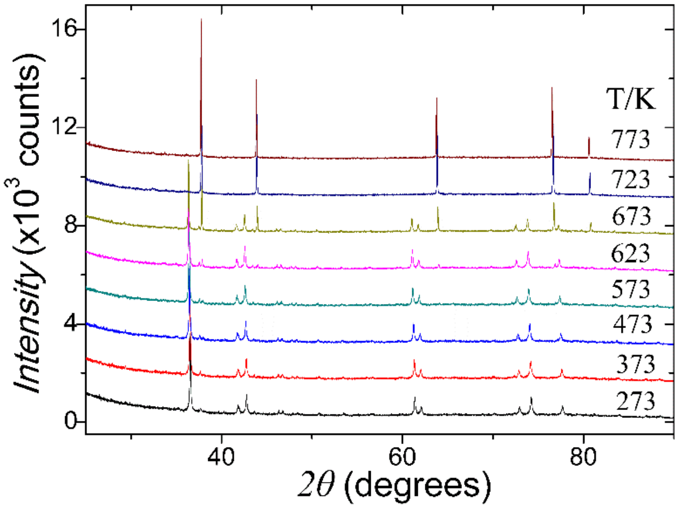


Figure S1. Temperature dependent powder X-ray diffraction indicating $Ag_{16}B_4O_{10}$ start to decompose into elemental Ag at about 623 K

**Table S1.** Crystal data, data collection and refinement details for $Ag_{16}B_4O_{10}$ at 298 K.

| | |
|---|---|
| Empirical formula | $Ag_{16}B_4O_{10}$ |
| Formula weight | 1929.16 |
| Space group (no.), *Z* | $I4_1/a$ (88), 4 |
| Lattice parameters /Å, | $a$ = 13.3481(5)<br>$c$ = 8.6228(4) |
| $V$ /Å$^3$ | 1536.3(1) |
| $\rho_{xray}$ /g×cm$^{-3}$ | 8.340 |
| Crystal size /mm$^3$ | 0.12 × 0.08 × 0.06 |
| Diffractometer | SMART APEX I, Bruker AXS |
| X-ray radiation, $\lambda$/Å | MoK$_\alpha$, 0.71073 |
| Absorption correction | Multi-scan, SADABS |
| $2\theta$ range /° | $5.62 \leq 2\theta \leq 103.92$ |
| Index ranges | $-29 \leq h \leq 29$, $-27 \leq k \leq 29$, $-19 \leq l \leq 17$ |
| Reflections collected | 31920 |
| Data, $R_{int}$ | 4280, 0.032 |
| No. of parameters | 170 |
| Transmission: $t_{min}$, $t_{max}$ | 0.198, 0.381 |
| Final R indices [I > 2$\sigma$(I)] | R1 = 0.033, wR2 = 0.080 |
| R indices (all data) | R1 = 0.037, wR2 = 0.082 |
| Deposition no. | CSD- 1951872 |

**Table S2.** Atomic coordinates and displacement parameters $U_{eq}$ /$10^{-4}$ Å$^2$.

| Atom | Site | *x* | *y* | *z* | $U_{eq}$ |
|---|---|---|---|---|---|
| Ag1 | 16*f* | 0.16299(2) | 0.54868(2) | 0.02757(2) | 135.8(3) |
| Ag2 | 16*f* | 0.25826(2) | 0.73949(2) | 0.01031(2) | 136.7(3) |
| Ag3 | 16*f* | 0.35583(2) | 0.45035(2) | 0.00220(2) | 140.7(3) |
| Ag4 | 16*f* | 0.10879(2) | 0.69713(2) | 0.25948(2) | 148(3) |
| O1 | 8*e* | 0 | ¾ | 0.0738(2) | 102(2) |
| O2 | 16*f* | 0.1208(1) | 0.7899(1) | –0.1258(2) | 103(2) |
| O3 | 16*f* | 0.3371(1) | 0.3329(1) | 0.1788(2) | 123(2) |
| B | 16*f* | 0.4160(2) | 0.2933(2) | 0.2730(3) | 108(3) |

**Table S3.** Selected interatomic distances /Å and angles /°.

| Atomic contact | Distance /Å | Atomic contact | Angle /° |
|---|---|---|---|
| Ag1 — O2 | 2.350(2) | O1 — B — O2 | 106.4(2) |
| — O3 | 2.264(2) | | 107.6(2) |
| Ag2 — O2 | 2.280(1) | O1 — B — O3 | 111.9(2) |
| — O3 | 2.307(2) | O2 — B — O2 | 107.4(2) |
| Ag3 — O3 | 2.200(2) | O2 — B — O3 | 111.3(2) |
| Ag4 — O1 | 2.274(1) | | 111.9(2) |
| B — O1 | 1.512(3) | | |
| — O2 | 1.485(3) | | |
| — O2 | 1.496(3) | | |
| — O3 | 1.431(3) | | |

Table S4. Refined atomic parameters against PXRD in space group I $4_1$/a:2. The refined lattice parameters are a = 13.3614(4) Å and c =8.6288(3) Å

| Atom | Site | *x* | *y* | *z* |
|---|---|---|---|---|
| Ag1 | 16*f* | 0.1633(3) | 0.5497(2) | 0.0265(3) |
| Ag2 | 16*f* | 0.2578(3) | 0.7394(2) | 0.0139(4) |
| Ag3 | 16*f* | 0.3550(2) | 0.4506(3) | 0.0005(3) |
| Ag4 | 16*f* | 0.1087(2) | 0.6966(3) | 0.2607(5) |
| O1 | 8*e* | 0 | ¾ | 0.0738 |
| O2 | 16*f* | 0.1208 | 0.7899 | –0.1258 |
| O3 | 16*f* | 0.3371 | 0.3329 | 0.1788 |
| B | 16*f* | 0.4160 | 0.2933 | 0.2730 |

## Computational details

## Pseudopotentials (scalar relativistic) and basis sets

| | **Ag** | | **B** | | **O** | |
|---|---|---|---|---|---|---|
| Pseudopotential<br>core<br>valence<br>ref. | <br>[Ar]$3d^{10}$<br>$4s^24p^64d^{10}5s^1$<br>[S1] | | <br>[He]<br>$2s^22p$<br>[S2] | | <br>[He]<br>$2s^22p^4$<br>[S2] | |
| | exponent | coefficient | exponent | coefficient | exponent | coefficient |
| s-shell | 9.088442<br>7.540731<br>2.794005<br><br>1.480158<br><br>0.653851<br><br>0.35 | -1.9648132<br>2.7332194<br>0.1991148<br><br>1.0<br><br>1.0<br><br>1.0 | 1.690560<br>0.983666<br>0.256979 | -0.272208<br>0.201128<br>0.577763 | 47.105518<br>5.911346<br>0.976483<br><br>0.296070 | -0.014408<br>0.129568<br>-0.563118<br><br>1.0 |
| p-shell | 4.451240<br>3.675263<br><br>1.291288<br>0.652578<br><br>0.36704 | -6.083378<br>6.4168543<br><br>0.7539735<br>0.2730597<br><br>1.0 | 5.399913<br>1.271217<br>0.361909 | 0.034941<br>0.186834<br>0.468463 | 16.692219<br>3.900702<br>1.078253<br><br>0.284189 | 0.044856<br>0.222613<br>0.500188<br><br>1.0 |
| sp-shell | 0.18 | 1.0 1.0 | 0.12 | 1.0 1.0 | 0.12 | 1.0 1.0 |
| d-shell | 7.99473<br>2.784773<br>1.209744<br>0.505393<br><br>0.198851 | -0.0163876<br>0.2814107<br>0.4863264<br>0.3867258<br><br>1.0 | 0.5 | 1.0 | 1.2 | 1.0 |

## Tolerance parameters used in the CRYSTAL17 input:

TOLINTEG 12 12 12 12 24

TOLPSEUD 12

TOLDEE 8

BIPOLAR 128 128

## Structural parameters used in the calculation

| Lattice | |
|---|---|
| a / Å<br>c / Å | 13.3481<br>8.6228 |
| Site parameter | |
| Ag1<br>Ag2<br>Ag3<br>Ag4<br>O1<br>O2<br>O3<br>B | 0.16199 0.54868 0.02757<br>0.25826 0.73949 0.01031<br>0.35583 0.45035 0.00220<br>0.10879 0.69713 0.25948<br>0.00000 0.75000 0.07380<br>0.12081 0.78993 -0.12579<br>0.33712 0.33285 0.17879<br>0.41600 0.29325 0.2730 |


[S1] Andrae, D.; Haeussermann, U.; Dolg, M.; Stoll, H.; Preuss, H. Energy-adjusted *ab initio* pseudopotentials for the second and third row transition elements. *Theor. Chim. Acta*, **1990**, *77*, 123-141.

[S2] Bergner, A.; Dolg, M.; Küchle, W.; Stoll, H.; Preuss, H. *Ab initio* energy-adjusted pseudopotentials for elements of groups 13–17, *Mol. Phys.* **1993**, *80*, 1431-1441.
